# Draft Genome Sequence of Riemerella anatipestifer Strain xi1

**DOI:** 10.1128/mra.01034-21

**Published:** 2022-03-16

**Authors:** Xiaona Wei, Zhuanqiang Yan, Qingfeng Zhou

**Affiliations:** a Guangdong Enterprise Key Laboratory for Animal Health and Environmental Control, Wen’s Foodstuff Group Co. Ltd., Yunfu, China; b Wen’s Group Academy, Wen’s Foodstuff Group Co., Ltd., Xinxing, Guangdong, China; University of Maryland School of Medicine

## Abstract

Riemerella anatipestifer is an important bacterial pathogen associated with epizootic infections in waterfowl and various other birds. The complete genome sequence of Riemerella anatipestifer xi1, isolated in China, was sequenced. The genome consisted of 2,062,911 bp carrying 1,879 predicted genes.

## ANNOUNCEMENT

Riemerella anatipestifer, a member of the genus *Riemerella* in the family *Flavobacteriaceae*, mainly infects domestic ducks, geese, turkeys, and other birds, causing pericarditis, perihepatitis, and meningitis (1). In the duck industry, R. anatipestifer is a major cause of bacterial infections, and these infections cause considerable economic loss (2). A total of 21 serotypes of R. anatipestifer, with little or no cross-protection among them, have been identified (3). A limited number of genomic resources of R. anatipestifer are available (3–7). R. anatipestifer strain xi1 was isolated from dead ducks. Briefly, liver tissues were collected by inoculation ring and coated on Trypticase soy agar (TSA) tablets containing 5% bovine serum and cultured overnight at 37°C in an atmosphere of 5% CO_2_. Single colonies were selected for further R. anatipestifer identification, including Gram stain, specific PCR amplification, and biochemical identification. The primers used in PCR identification are listed in Table 1 (8, 9). Briefly, bacterial solutions were heated at 100°C for 5 min and centrifuged at 8,000 to ∼9,000 rpm/min for 5 min, and the supernatants containing bacterial genome were collected as the DNA template. The PCR reaction system consisted of 10 μL of 2× *Taq* mix enzyme, 1 μL of each primer, 1 μL of template, and 5 μL of double-distilled water (ddH_2_O), with the following procedure: 94°C predenaturation for 3 min, 94°C denaturation for 30 s, 55°C annealing for 30 s, 72°C extension for 1 min, a total of 35 cycles, and then extension at 72°C for 10 min and holding at 4°C. Two specific bands at 644 bp and 194 bp were obtained after amplification. Biochemical testing was carried out with bacterial microbiochemical reaction tubes (Hangzhou Microbial Reagent Co., Ltd., China) following the manufacturer’s instructions, in which the isolated strains could use oxidase, urease, and contact enzyme, could not use carbohydrates, citrate, urea, arginase, and lysine enzyme, and could not produce H_2_S and indole. The identified strain, named xi1, was cultured in TBS containing bovine serum in an atmosphere of 5% CO_2_ for 16 h at 37°C for genome extraction. The genome was prepared and sequenced using an Illumina NovaSeq 6000 instrument (Magigene, China) in paired-end (2 × 150 bp) format.

**TABLE 1:** Primers used to identify the isolated strains

Gene	Primer^*a*^	Primer sequence (5′∼3′)	Product size (bp)
*gyrb*	F	AGAGCGAGAAGAAAAAACCT	194
	R	CTCCCATAAGCATAGAGAAGA	
190	F	GTATTGAAAGCTCTGGCGG	644
843	R	TCGCTTAGTCTCTGAACCC	

a F, forward; R, reverse.

After sequencing, 3,269,733 raw reads were obtained and subjected to extraction of host data. A total of 1,981,433 raw reads were preprocessed for quality checking using FastQC version 0.11.9 (10) and further assembled using SOAPdenovo version 2.04 (11). The genome coverage was 350×. The final genome assembly was represented by 173 scaffolds (>500 bp), the total length was 2,062,911 bp, and the maximum length was 87,505 bp. The G+C content was 35.14%. The gene annotation was performed using the Prokaryotic Genome Annotation Pipeline (PGAP) version 5.3 (12), and a total of 2,021 genes were predicted; among these, 1,958 genes encode proteins. The average length of the genes was 955 bp. The genome contained 193 interspersed repeated sequences (INSs) and 89 tandem repeats (TRs). In addition, 34 tRNAs were obtained using tRNAscan-SE version 1.3.1 (13). Among the 2,021 genes, 1,089 and 1,305 genes were assigned to KEGG pathways and Clusters of Orthologous Genes (COG) families, respectively. Default parameters were used for all software unless otherwise specified.

Overall, the genome sequence of avirulent R. anatipestifer xi1 will provide the genetic background for understanding the pathogenicity mechanisms of this pathogen using comparative genomics.

### Data availability.

This whole-genome shotgun project has been deposited at DDBJ/ENA/GenBank under the accession number JAJDFB000000000. The version described in this paper is version JAJDFB010000000. The BioProject number is PRJNA772301, and the SRA accession number is SRR16620534.
